# Tissue Microarray Immunohistochemical Detection of Brachyury Is Not a Prognostic Indicator in Chordoma

**DOI:** 10.1371/journal.pone.0075851

**Published:** 2013-09-23

**Authors:** Linlin Zhang, Shang Guo, Joseph H. Schwab, G. Petur Nielsen, Edwin Choy, Shunan Ye, Zhan Zhang, Henry Mankin, Francis J. Hornicek, Zhenfeng Duan

**Affiliations:** 1 Sarcoma Biology Laboratory, Center for Sarcoma and Connective Tissue Oncology, Massachusetts General Hospital, Boston, Massachusetts, United States of America; 2 Department of Pathology, the Third Affiliated Hospital of Zhengzhou University, Zhengzhou, China; 3 Department of Pathology, Massachusetts General Hospital, Boston, Massachusetts, United States of America; 4 Department of Orthopaedics, Shanghai Sixth People’s Hospital affiliated to Shanghai Jiao Tong University, Shanghai, China; Van Andel Institute, United States of America

## Abstract

Brachyury is a marker for notochord-derived tissues and neoplasms, such as chordoma. However, the prognostic relevance of brachyury expression in chordoma is still unknown. The improvement of tissue microarray technology has provided the opportunity to perform analyses of tumor tissues on a large scale in a uniform and consistent manner. This study was designed with the use of tissue microarray to determine the expression of brachyury. Brachyury expression in chordoma tissues from 78 chordoma patients was analyzed by immunohistochemical staining of tissue microarray. The clinicopathologic parameters, including gender, age, location of tumor and metastatic status were evaluated. Fifty-nine of 78 (75.64%) tumors showed nuclear staining for brachyury, and among them, 29 tumors (49.15%) showed 1+ (<30% positive cells) staining, 15 tumors (25.42%) had 2+ (31% to 60% positive cells) staining, and 15 tumors (25.42%) demonstrated 3+ (61% to 100% positive cells) staining. Brachyury nuclear staining was detected more frequently in sacral chordomas than in chordomas of the mobile spine. However, there was no significant relationship between brachyury expression and other clinical variables. By Kaplan-Meier analysis, brachyury expression failed to produce any significant relationship with the overall survival rate. In conclusion, brachyury expression is not a prognostic indicator in chordoma.

## Introduction

Chordoma is a rare malignant bone tumor but is the most common primary malignant tumor of the mobile spine and the sacrum [1,2]. Chordoma is slow growing and is often detected only after substantial growth [2,3]. Due to location, they are difficult to treat and have a high local recurrence rate; furthermore, no biomarkers are available to predict their clinical behavior.

Chordoma is thought to be derived from notochordal remnants or so called benign notochordal cell tumors (BNCT) [2,3]. However, the carcinogenesis and pathogenesis for development of chordoma remains largely unclear, although studies have showed that the gain of brachyury locus is common in chordomas, and expression of this gene might play a crucial role in the pathogenesis of chordoma [4-7]. Brachyury (also known as transcription factor T) is a member of the T-box family, expressed commonly in notochord cells and plays a pivotal role in notochord development and formation [8]. Expression of brachyury is regulated by the Wnt signaling pathway which in turn is mediated by the β-catenin/TCF4 complex in the development of mesoderm tissues in mouse [9,10]; however, active WNT signalling has not been established in chordoma. Brachyury is also significantly expressed in the majority of chordoma tissues in comparison to other types of cancer and thought to be a novel biomarker for chordoma [4,6,11]. High-resolution array comparative genomic hybridization (CGH) shows unique duplications in the 6q27 region in tumor samples from patients with familial chordoma [5]. In sporadic chordoma, a study of 21 tumors analyzed by CGH showed large copy number losses, involving chromosomes 1p, 3, 4, 9, 10, 13, 14, and 18, were more common than copy number gains. Most of these sporadic tumors are not associated with brachyury duplication or amplification [12]. In comparison, another study noticed that copy number gain (CNG) of brachyury gene was found in 92 out of 170 chordoma patients by fluorescence in situ hybridization (FISH) [13]. However, the association between the protein level of brachyury expression and clinical behavior in chordoma is still unknown.

The tissue microarray (TMA) is a recently implemented, high-throughput technology used in the analysis of molecular markers in oncology. As compared with conventional immunohistochemistry, TMAs allow for a large scale study of tumor tissue samples in a uniform and consistent manner [14-16]. . For this study we designed a chordoma TMA which included tumor tissues from 78 patients to correlate the expression of brachyury with clinical outcome.

## Materials and Methods

### Ethic Statement

The study protocol and the consent of the informed patients were approved by the Partners Human Research Committee (number: 2007-P-002464/5 valid until 10/19/2013). All patients were informed of their information being stored in the hospital database and used for research and have given their written approval.

### Patient’s Clinical Data and Specimens

A retrospective study of 78 chordoma patients was identified for TMA immunohistochemical staining by using the Massachusetts General Hospital cancer registry and orthopedic oncology databases. The data of each patient’ age, gender, date of birth, tumor location(s), date of death (if applicable), and disease status were collected. Those patients with archival tissue blocks available through the Department of Pathology were selected. The hematoxylin-eosin (H&E) stained slides of the paraffin-embedded tumor specimens were reviewed by one of the authors (GPN). In this study, we only inculded chordomas with a conventional morphology, whereas dedifferentiated and chondroid chordoma subtypes were excluded. The clinical data of chordoma patients were presented in the Table 1.

**Table 1:** The clinical parameters of chordoma tissue microarray.

Parameters		n(%)
Age		46
	<45	8 (17.40)
	45-60	16 (34.78)
	>60	22 (47.83)
Gender		74
	Male	56 (75.67)
	Female	18 (4.32)
Location		63
	Mobilespine	21 (33.33)
	Sacrum	42 (66.67)
Prognosis		61
	Survival	27 (44.26)
	Nonsurvival	34 (55.73)
Disease status		78
	Primary	29 (37.17)
	Recurrence	41 (52.56)
	Metastasis	8 (10.25)
Disease status		78
	NED	27 (44.26)
	AWD	7 (11.47)
	DOD	23 (37.7)
	Dead with other disease	4 (6.55)

### Construction of the Chordoma TMA

Representative areas of chordoma tumor slides of the paraffin-embedded tumor specimens for each case were selected and circled to match the blocks for the TMA. The blocks matching the circled slides were retrieved to prepare the recipient block for the microarray. To ensure accurate representation of the selected cores, three areas of tumor parts per case were selected for assembling the recipient master blocks. Each target area on the selected blocks was punched to form a 0.5 mm diameter tissue core and was placed consecutively on the recipient master blocks. The chordoma TMA was constructed by the Tissue Microarray and Imaging Core at the Dana-Farber/Harvard Cancer Center (http://genepath.med.harvard.edu:8080/pathcore/).

### Immunohistochemical Staining and Analysis

A tissue microarray of chordoma was used for this retrospective analysis, including 291 cores from 97 specimens (78 chordoma cancers, 6 notochord controls, 2 chordoma cell lines (UCH1 and CH 8) [17] and 11 tissue controls including 7 normal tissues near the chordoma, 4 other malignant tumors (each of breast, kidney, melanoma and liver cancer) Immunohistochemical stain with goat antibody (sc-17745) against human Brachyury (Santa Cruz Biotechnology, Inc., CA, USA) was performed by using Cell and Tissue Staining Kit (Goat kit, R&D Systems, Inc., Minneapolis, MN, USA). Briefly, 5-µm-thick array sections were baked at 60°C for 1h, dewaxed with xylene (triple for 5 minutes), transferred through 100% ethanol (twice for 5 minutes), rehydrated through graded alcohol, and then immersed in deionized water for 10 minutes. Antigen retrieval was processed with Target Retrieval Solution (Dako, North America, Inc., CA, USA) following the instruction of the manufacturer. In brief after antigen retrieval, the slide was washed with PBS twice for 5 minutes. Endogenous avidin/biotin binding was blocked according to the instruction of staining kit. Primary goat brachyury antibody was applied at 4 °C overnight (1:100 dilution, in 1% bovine serum albumin PBS). After incubation with the biotinylated anti-goat antibody, and then with HSS-HRP, the slide was rinsed in PBS thrice, bound antibody was detected with the substrate reagents from HRP-DAB system of Cell and Tissue Staining Kit. Finally, sections were counterstained with Hematoxylin QS (Vector Laboratories) and the slide was mounted with VectaMount AQ (Vector Laboratories).

Brachyury-positive samples were defined as those showing nuclear staining. Brachyury staining patterns were categorized into 4 groups: 0, no nuclear staining; 1+, <=30% staining of tumor cells; 2+, 31% to 60% staining of tumor cells; 3+, 61% to 100% staining of tumor cells. The percentage of cells showing positive nuclear staining for brachyury was calculated by reviewing the entire spot. Categorizing the brachyury staining was completed by 2 independent investigators in the Sarcoma Research Laboratory, who were blinded to the clinicopathologic data, with a consensus reached in all cases. Brachyury staining images were obtained by using a Nikon Eclipse Ti-U fluorescence microscope (Nikon Corp) with a SPORT RT digital camera (Diagnostic Instruments Inc.).

### Statistical Analyses

A two-sided Student’s t-test (GraphPad PRISM Software; GrahPad Software, Inc., CA, USA) was used to compare the differences between groups, including male vs. female, survival vs. non-survival, and mobile spine vs. sacrum. Analyses were also performed by comparing the relationship between the expression of brachyury and age, metastasis of tumor and other disease status. Results are given as mean + SD and values with P < 0.05 were considered as statistically significant. Kaplan–Meier survival analysis was used to analyze the correlation between the level of brachyury expression and prognosis. Survival time was calculated from the date of tumor diagnosis to the date of death or last follow-up.

## Results

### Expression of Brachyury in Chordoma

**Figure 1:**
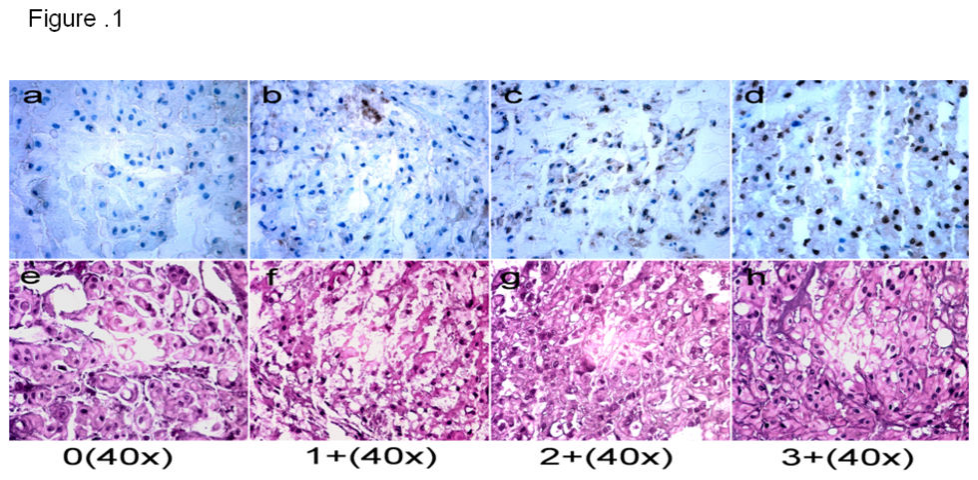
Representative expression of brachyury in chordoma tissues on TMA slide. **a** 0 staining, no nuclear staining of tumor cells; **b** 1+ staining, <=30% nuclear staining of tumor cells, **c** 2+ staining 31% to 60% positive nuclear staining; and **d** 3+ staining, 61% to 100% nuclear staining.

Brachyury nuclear staining was detected in 59 (75.64%) of 78 tumors. Nineteen (24.35%) of 78 tumors were negative for brachyury staining (Figure 1a). Among the positive tumors, 29 (49.15%) showed 1+ (<=30% of positive nuclear staining cells) staining (Figure 1b), 15 tumors (25.42%) had 2+ (31% to 60% of positive nuclear staining cells) staining (Figure 1c), and 15 tumors (25.42%) demonstrated 3+ (61% to 100% of positive nuclear staining cells) staining (Figure 1d).

In addition, 6 notochord controls and 4 other malignant tumors, breast, kidney, melanoma and liver cancer were positive for brachyury nuclear staining. One of 2 chordoma cell lines (UCH1) also stained positive for brachyury. Normal liver tissue did not demonstrate nuclear expression of brachyury.

### Association of Brachyury Expression and Clinical Data

Based on the location of chordoma, we grouped the chordomas into two groups, those arising in the mobile spine and those arising in the sacrum. There was a significant difference in the expression of brachyury between tumors of the mobile spine and those arising in the sacrum (_*P*_=0.027) (Table 2); brachyury nuclear staining was detected more frequently in sacral chordomas than in mobile spine chordomas. Chordoma patients were also divided into 2 groups based on gender and 3 groups based on their age (less or equal than 45 years old group, 46 to 60 years old group and over 60 years old group). Immunohistochemical staining showed that expression of brachyury was not associated with gender or age (Table 2). With respect to the relationship between expression of brachyury and disease status, brachyury nuclear staining was present in 23 of 29 (79.31%) primary chordomas, 31 of 41 (75.60%) recurrent chordomas and 5 of 8 (62.5%) metastatic chordomas. No significant difference of brachyury expression was detected between primary and recurrent chordoma (_*P*_=0.69), or primary and metastatic chordoma (_*P*_=0.37) (Table 2).

**Table 2:** Brachyury immunohistochemical staining scores in different groups of chordomas.

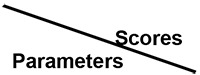	**0**	**1+**	**2+**	**3+**	***P* value**
Gender					
Male	13	22	11	10	0.69
Female	4	6	4	4	
Location					
Mobilespine	9	7	2	3	0.03
Sacrum	6	16	10	10	
Prognosis					
Survival	6	10	6	5	0.84
Nonsurvival	9	12	6	7	
Age					
subgroup 1: <45	3	3	1	1	0.41^*^
subgroup 2: 45-60	4	4	6	2	0.80^#^
subgroup 3: >60	4	9	3	6	0.28^&^
Disease status					
subgroup 1: Primary	6	12	3	8	0.69^*^
subgroup 2: Recurrence	10	15	9	7	0.50^#^
subgroup 3: Metastasis	3	2	3	0	0.37^&^

* indicates the P value of subgroup 1 v.s. subgroup 2

# indicates the P value of subgroup 2 v.s. subgroup 3

& indicates the P value of subgroup 1 v.s. subgroup 3

### Expression of Brachyury and Prognosis

**Figure 2:**
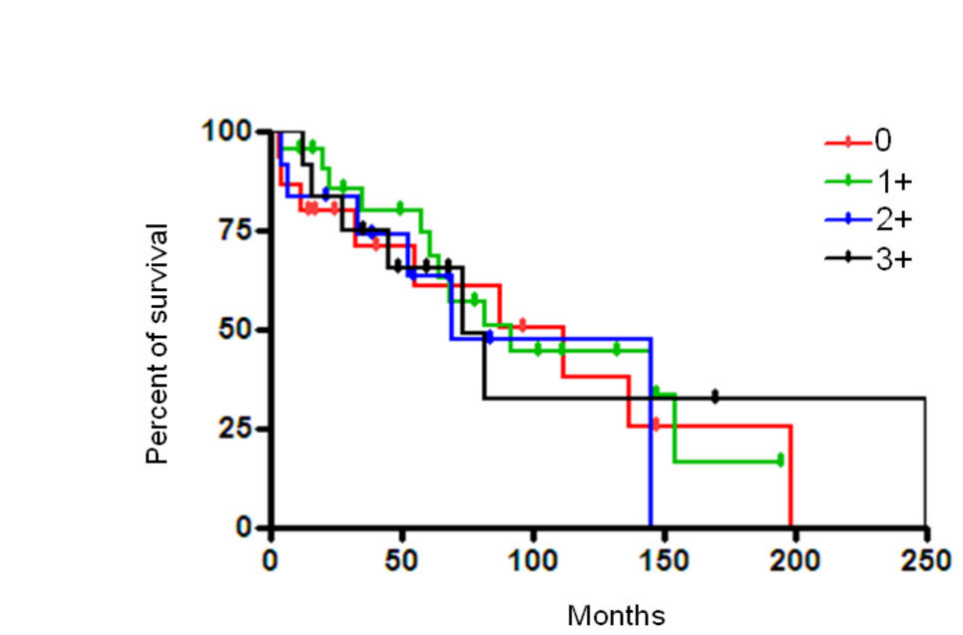
Analyses of association between expression of brachyury and survival for chordoma. Kaplan-Meier survival analysis showed that the expression of brachyury was not associated with prognosis in patients with chordoma.

Follow-up data were available for 61 of 78 (78.20%) patients. The median follow-up period was 68.70 (range from 3.63 to 249.63) months. At the date of last follow-up, 27 patients had died of disease (DOD), 7 patients were alive with disease (AWD), 23 patients had no evidence of disease (NED) and 4 patients had died of other causes. No significant difference in brachyury expression was identified between these groups of patients (data not shown). By Kaplan-Meier survival analysis, brachyury expression failed to have any significant relationship with the overall survival rate for chordoma patients (Figure 2). There was no significant difference between survival and non-survival groups (_*P*_=0.8479, Table 2).

## Dissusion

New technological developments such as TMAs are extremely useful for the study of a large cohort of tumor samples. TMA is an effective tool, particularly in translational research and clinical trials, allowing resource-efficient use, and high-throughput profiling of large numbers of tumors, although a concern has been raised about the representation of tumor tissues on the TMA to the whole tumor [14,15]. However, this potential problem can be overcome by accurately evaluating the original tumor tissues collected from a highly representative area, together with the inclusion of three cores in assembling the TMA. In addition, the evaluation of a large cohort of tumor samples in a single slide is very valuable, especially in an extremely rare tumor such as chordoma in which approximately 300 cases are diagnosed each year in the United States (http://www.chordomafoundation.org). Therefore, we constructed a chordoma TMA with 78 tumor samples, 6 notochord controls, 2 chordoma cell lines and 11 tissues controls including 7 normal tissues adjacent to the chordoma and 4 other malignant tumors.

Recent reports suggest that brachyury might be essential for the survival or proliferation of tumor cells [4,6]. Brachyury is a gene of emerging significance in cancer; it is duplicated in individuals with familial chordoma and it is amplified in around 7% of sporadic chordomas, and silencing of the gene in vitro by siRNA induces growth arrest of chordoma cells [6]. The results of immunohistochemical examinations of brachyury are controversial. Several studies have shown that brachyury is not expressed in non-chordoma tumors but recent studies have shown that brachyury is expressed in many other types of tumors, including hemangioblastoma, breast, bladder, kidney, ovary, prostate, colon and lung cancers [4,18-20]. Reports have shown that brachyury is expressed in 41% of primary lung carcinomas, including 48% of adenocarcinomas and 25% of squamous cell carcinomas [18,19]. A more recent study found *in vivo* treatment of tumor xenografts of human lung carcinoma cells with chemotherapy results in the selective growth of resistant tumors with high levels of brachyury protein expression [21]. Therefore, targeting brachyury may offer new therapeutic options for treating various cancers, including chordoma [22]. In order to characterize the relationship between the protein level of brachyury expression and clinical behavior, we used a chordoma TMA that allowed the simultaneous characterization of the expression status of brachyury in 78 chordoma samples. Our study showed that 59 of 78 (75.64%) tumors had positive brachyury nuclear staining. Among them, 29 tumors (49.15%) had 1+ (<=30% of positive nuclear staining cells) staining, 15 tumors (25.42%) had 2+ (31% to 60% of positive nuclear staining cells) staining, and 15 tumors (25.42%) showed 3+ (61% to 100% of positive nuclear staining cells) staining. In comparison, previous studies have shown brachyury expression in 80 to 90% of chordoma [4,23,24]. This discrepancy between reported results and our TMA staining are most likely due to differences in the relative sensitivities of the methods used such as different antigen retrieval procedure, or heterogeneity of the material analyzed. Moreover, the immunohistochemical staining of chordoma in most previous studies is based on multiple slides, rarely on a single TMA slide.

Statistical analysis showed that brachyury nuclear staining was detected more frequently in sacral chordoma than in mobile spine chordoma. The reason for brachyury positive staining in sacral chorndoma is higher than middle spine is unknown, but may reflect the location of notochord remanants, the main embryonic axial structure, which is thought to be the origin of where chordoma is derived.

It has been reported that early stages colorectal cancer patients showed decreased survival when brachyury was expressed in the tumor tissue, while no correlation was observed in patients with later tumor stages [25]. In the current study, brachyury expression failed to exhibit a significant relationship with the overall survival rate for chordoma patients. There was no significant relationship between brachyury expression and other clinical variables.

In conclusion, this study showed brachyury expression is not associated with the clinical behavior in chordoma. Further studies of a larger sample size and additionally chordoma biomarkers that could predict clinical outcome are needed.
